# Applications of BiOX in the Photocatalytic Reactions

**DOI:** 10.3390/molecules28114400

**Published:** 2023-05-28

**Authors:** Zhimin Yuan, Zaiyong Jiang

**Affiliations:** School of Chemistry & Chemical Engineering and Environmental Engineering, Weifang University, Weifang 261061, China

**Keywords:** photocatalytic CO_2_ reduction, photocatalysts, BiOX (X = Cl, Br, I), photocatalytic reactions, layer structure

## Abstract

BiOX (X = Cl, Br, I) families are a kind of new type of photocatalysts, which have attracted the attention of more and more researchers. The suitable band gaps and their convenient tunability via the change of X elements enable BiOX to adapt to many photocatalytic reactions. In addition, because of their characteristics of the unique layered structure and indirect bandgap semiconductor, BiOX exhibits excellent separation efficiency of photogenerated electrons and holes. Therefore, BiOX could usually demonstrate fine activity in many photocatalytic reactions. In this review, we will present the various applications and modification strategies of BiOX in photocatalytic reactions. Finally, based on a good understanding of the above issues, we will propose the future directions and feasibilities of the reasonable design of modification strategies of BiOX to obtain better photocatalytic activity toward various photocatalytic applications.

## 1. Introduction

With the rapid development of the economy and society, the consumption of underground fossil energy, such as oil and coal, is increasing rapidly [1]. The energy crisis has gradually become the main factor restricting economic development. On the other hand, the consumption of fossil energy produces serious environmental problems, such as air and water pollution, the greenhouse effect, fog, and so on [2,3]. To deal with the energy crisis and environmental pollution, scientists worldwide are exploring a solution. Among them, semiconductor photocatalysis is a promising solution [4,5,6,7,8,9,10]. These semiconductor photocatalysts can absorb solar light to split H_2_O to produce H_2_ and O_2_ gas [11], reduce carbon dioxide to organic carbon resources (such as methane, carbon monoxide, methanol, etc.) [12,13,14], degrade organic pollutants [15], reduce nitrogen to ammonium ion [16], reduce heavy metal ions [17], kill bacteria [18], and so on. As can be seen from the above description, photocatalysis can generate environmentally friendly clean energy only by using sunlight and solve the serious environmental pollution problem [19,20]. Therefore, it is regarded as a promising method to solve energy and environmental problems [21,22]. At present, the semiconductor photocatalysis has become a research hotspot. 

Up to now, many photocatalysts have been discovered and exhibited excellent photocatalytic activities, such as TiO_2_ [23], ZnO [24], ZrO_2_ [25], In_2_O_3_ [26], BiOX [27,28,29], C_3_N_4_ [30], MOF [31], etc. Among them, BiOX (X = Cl, Br, I) families are a newly discovered type of photocatalysts, which is the crystal structure of PbFCl type (space groups P4/nmm, D_4h_ symmetry tetragonal system) [32,33]. Therefore, the BiOX crystal structure belongs to a layered type, which are composed of the staggered arrangement of the [Bi_2_O_2_]^2+^ layer and the double X atom layer in the direction of the c-axis [34,35]. Additionally, the layers of [X-Bi-O-Bi-X] are connected by the weak van der Waals interaction [36,37]. Enough space can be provided between layers, which favors polarizing related atoms and orbitals to produce an internal electric field between the [Bi_2_O_2_]^2+^ layer and the X atom layer. The formed internal electric field could promote the separation of photogenerated electrons and hole pairs, which is very important for the photocatalytic activities of BiOX [38,39]. In addition, as the indirect band gap semiconductors, the photo-generated electrons derived from the valence band of BiOX need to pass a certain k-space distance to transfer the conduction band, which could lead to a low recombination rate of photon-generated carriers [40,41]. Because of their characteristics of the unique layered structure and indirect bandgap semiconductor, BiOX could usually demonstrate fine activity in many types of photocatalytic reactions. 

To date, there are some review papers reported on the applications of BiOX in photocatalysis, but they are mostly focused on a particular area, such as organic degradation, CO_2_ reduction, etc [42,43,44,45,46]. However, few of them have summarized and reported the comprehensive applications of BiOX in the whole field of photocatalysis. To make it easier and more convenient for most researchers to fully understand the applications of BiOX in photocatalytic reactions in the future, it is necessary to conduct a more comprehensive application review. In this review, we summarize relatively comprehensive recent applications of BiOX in photocatalytic reactions. We will summarize and analyze these applications in the following six fields (Table 1): BiOX split H_2_O to produce H_2_ and O_2_ gas, degrade organic pollutants, photocatalytic nitrogen fixation, degrade the inorganics (hexavalent chromium ions), reduce carbon dioxide to organic carbon resources (such as methane, carbon monoxide, methanol, etc.), and kill bacteria. This review will be completed by presenting the mechanism of application of BiOX in each field, progress, and some modification strategies to further enhance their activities, and some current problems or challenges and future research directions.

## 2. The Difference of Band Gap among BiOX Series Photocatalysts

In photocatalytic reactions, the band gap of the photocatalyst is very important. As far as we know, the band gaps of BiOX are about 3.2 (BiOCl), 2.6 (BiOBr), and 1.7 (BiOI) eV, respectively [47]. Especially the BiOBr and BiOI, with good visible light absorption capacity, are very conducive to the efficient use of solar light in the photocatalytic reaction. Moreover, Zhao et al. [48] reported that the valence band consists mainly of O 2p and X np (Cl = 3p, Br = 4p and I = 5p, respectively), and the conduction band consists of Bi 6p states. We all know that Cl, Br, and I belong to the seventh main group elements, they should possess similar properties. Therefore, their band gaps might be conveniently tuned via the change of X elements. The suitable band gaps and convenient tunability enable BiOX to adapt to many photocatalytic reactions. Based on the previously-mentioned advantages, BiOX has attracted the attention of more and more researchers.

## 3. Recent Application of BiOX in the Field of Photocatalytic Reactions

### 3.1. Splitting H_2_O to Produce H_2_ and O_2_

Photocatalytic splitting of H_2_O began in 1972, when Fujishima and Honda discovered that H_2_O molecules could be split at a titanium dioxide electrode to produce hydrogen and oxygen under the photocatalysis, making it possible to use solar energy to split H_2_O into hydrogen [49]. After decades of exploration, many related photocatalysts have been discovered, such as TiO_2_, ZnO, CdS, ZnS, GaN, and so on [50,51,52,53,54]. In recent years, BiOX photocatalysts have also been applied to split H_2_O to produce hydrogen and oxygen gas, and some excellent results have been achieved [36,55,56,57,58,59,60].

**Figure 1:**
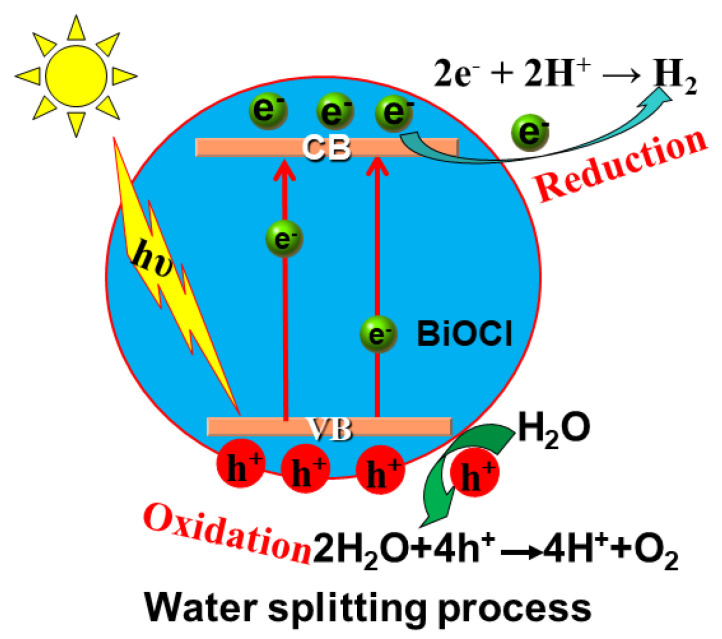
Schematic diagram of mechanism of the BiOCl photocatalytic splitting of H_2_O.

The kinetic realization of the BiOX photocatalytic splitting of H_2_O for hydrogen and oxygen production requires the following four processes (Figure 1) [61]: (1) absorption of photons by the BiOX photocatalyst to produce photogenerated electron-hole pairs; (2) the separation of photogenerated electron-hole pairs; (3) migration of photogenerated electrons and holes; and (4) surface chemical reactions. In other words, the electrons and holes migrating to the surface of the BiOX undergo reduction and oxidation reactions with water to produce H_2_ and O_2_, respectively. The chemical reaction mechanism can be simply expressed by the following reaction equation:

BiOX + hυ → e^−^ + h^+^ (Semiconductor excitation and Carriers separation.) (1)

2e^−^ + 2H^+^ → H_2_ (Half-reaction: photocatalytic hydrogen evolution.) (2)

4h^+^ + 2H_2_O → O_2_+ 4H^+^ (Half-reaction: photocatalytic hydrogen evolution.) (3)

H_2_O → H_2_ + 1/2O_2_ (Total reaction: photocatalytic splitting of H_2_O.) (4)

In addition, it is important to note that for BiOX to achieve photocatalytic splitting of whole H_2_O, their conduction band and valence band positions also need to meet certain requirements except having a suitable band gap. Their bottom level of the conduction band (CB) should be more negative compared to that of the redox potential of H^+^/H_2_ (H^+^/H_2_ = 0.00 V vs. SHE) [57]. On the other hand, the top level of the valence band (VB) need be more positive compared with the redox potential of O_2_/H_2_O (O_2_/H_2_O= +1.23 V, vs. SHE). However, the CB of BiOX is normally slightly more positive compared to that of H^+^/H_2_ potential level [61,62]. In this case, BiOX could not achieve photocatalytic splitting of H_2_O for hydrogen evolution. 

**Figure 2:**
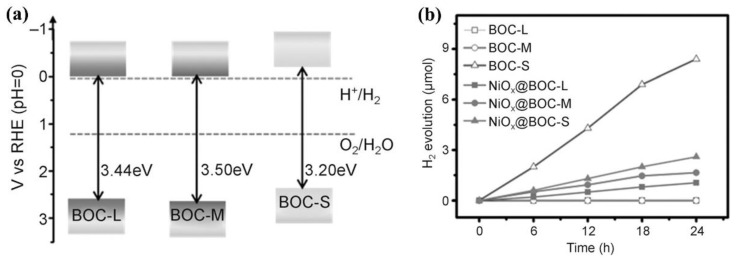
(**a**) Band-energy diagram of different BiOCl samples, (**b**) photocatalytic performance of splitting of H_2_O over different BiOCl samples. Reprinted with permission from Ref. [61]. Copyright 2015, Wiley.

Because BiOX have so many advantages, researchers are looking forward to applying it to the field of photocatalytic overall water splitting. Therefore, various modification strategies for BiOX are emerging in order to realize this idea. For example, Zhang et al. prepared BiOCl nanosheets as thick as quantum sizes (obtained sample was named BOC-S) [61], which can solve the conduction band (CB) position problem of this block BiOCl. As shown in Figure 2a, the position of CB of ultrathin BOC-S upshifted by −0.3 eV than that of BOC-L (BiOCl, ~200 nm thickness)/BOC-M (BiOCl, ~30 nm thickness). There are many bismuth vacancies and oxygen vacancies exist on the surface of ultrathin BiOCl nanosheets. In the process of photocatalytic overall water splitting, H_2_O adsorbed on the surfaces of BiOCl was transferred into an H atom and an OH group, subsequently, the OH group was trapped by the oxygen vacancy; On the other hand, the H atom bonds to a surface oxygen atom. The two H atoms on the surface of BiOCl could be dissociated to produce an H_2_ molecule because of the role of entropy. Finally, the ultrathin BiOCl nanosheet realized the photocatalytic overall water splitting without any sacrificial agents or co-catalysts (Figure 2b). In addition, it could be observed that both BOC-L and BOC-M did not have the photocatalytic performance of splitting H_2_O. These surface defects were proved to play an important role in the whole process via the first-principles calculations. In addition, Yang et al. prepared a kermesinus BiOI (K-BiOI) with many surface oxygen vacancies [63]. They found that the position of the CB edge of K-BiOI has extended to −0.41 V vs. SHE relative to that of common BiOI (0.26 V). The CB edge is more negative than the reduction potential of H^+^/H_2_, thereby realizing photocatalytic H_2_ production performance. To sum up, oxygen vacancy has a crucial impact on the BiOX photocatalyst to achieve photocatalytic overall water splitting.

### 3.2. Degrading Organic Pollutants

In 1976, Carey et al. carried out pioneering work in photocatalytic degradation of organic pollutants in water [64], opening the application field of photocatalytic technology in environmental protection, and then setting off a worldwide research boom in the emerging field of semiconductor photocatalytic degradation of organics. After nearly 50 years of efforts by scientists, there have been many successful applications of semiconductor photocatalytic oxidation technology. Many experimental facts have proved that semiconductor photocatalysis can remove various organic pollutants in the environment [65,66,67,68], such as alkanes, alkenes, phenols, a variety of simple aromatic compounds, and the corresponding halides, dyes, surfactants, herbicides, pesticides, humic acids, etc.

In recent years, BiOX catalysts have attracted extensive attention for their application in photocatalytic degradation of organics due to their unique electronic structure, highly anisotropic layered structure, good photocatalytic stability, cheap, and environmentally friendly [69,70,71]. When BiOX degrades organic pollutants, they are first photoexcited to produce photogenerated electrons and holes. Photogenerated electrons can react with O_2_ adsorbed on their surface to generate a series of free radicals with strong oxidation properties, such as O_2_🞄^−^, as shown in reaction Equations (5)–(10). Moreover, many photogenerated holes can directly react with H_2_O molecules or OH- ions adsorbent on BiOX surface to generate 🞄OH radical due to their strong oxidation ability. The reaction equation is shown in (11) and (12). Subsequently, these active free radicals with strong oxidation ability directly oxidize most organics into small inorganic molecules such as CO_2_ and H_2_O.

O_2_ + e^−^ → O_2_🞄^−^ (5)

H_2_O + O_2_🞄^−^ → HO_2_🞄 + OH^−^ (6)

2HO_2_🞄 → H_2_O_2_ + O_2_ (7)

H_2_O_2_ + e^−^ → 🞄OH + OH^−^ (8)

🞄OH + 🞄OH → H_2_O_2_ (9)

H_2_O_2_ + O_2_🞄^−^ → 🞄OH + OH^−^ + O_2_ (10)

H_2_O + h^+^ → 🞄OH + H^+^ (11)

OH^−^ + h^+^ → 🞄OH (12)

**Figure 3:**
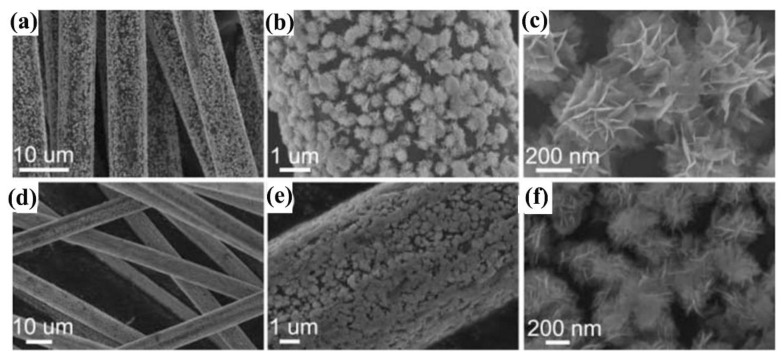
SEM images of BiOCl/ACF (**a**–**c**) and BiOCl/ACF (**d**–**f**). Reprinted with permission from Ref. [73]. Copyright 2015, The Royal Society of Chemistry.

For example, Yu et al. synthesized Bi/BiOCl nanosheets via one-step solution combustion synthesis [72]. They found that Bi/BiOCl-1 showed a 98% degradation efficiency of Rhodamine B (RhB) after visible light irradiation for 120 min. Through a series of studies, they proved that not only holes can directly react with RhB on the surface of BiOCl, but also O_2_🞄^−^ active species could also degrade the RhB to produce CO_2_ and H_2_O in the photocatalytic degradation process. In addition, in the current process of BiOX degradation of organics, the catalysts are still mainly concentrated in the powder state. Therefore, the recovery and reuse of BiOX powder catalysts after use is a great challenge in practical industrial applications. Therefore, our research group designed and loaded BiOCl and BiOBr onto the surface of activated carbon fiber via a facile solvothermal method and obtained BiOCl/ACF and BiOBr/ACF samples (Figure 3) [73]. Subsequently, the photocatalytic activity on decomposing RhB and 2,4-DCP aqueous solution has been tested using BiOCl/ACF and BiOBr/ACF samples. The experimental results exhibited that they had excellent cyclic properties and stable performance. This design successfully solved the difficult problem of their recovery and reuse.

Moreover, to further improve BiOX’s photocatalytic degradation activity of organics, many modification strategies have been proposed, such as heterojunction, element doping, crystal plane control, etc. [74,75,76]. Through the continuous exploration of scientific researchers, BiOX should have relatively good activities at degrading organics. However, the corresponding industrial application technology or recycling method still needs further exploration.

### 3.3. Photocatalytic Nitrogen Fixation

Currently, photocatalytic nitrogen fixation is considered one of the most ideal alternatives to the traditional Haber–Bosch nitrogen fixation method. Because photocatalytic nitrogen fixation reaction is carried out at room temperature and atmospheric pressure. Moreover, nitrogen is reduced by photogenerated electrons of the photocatalyst without consuming any fossil energy. This method directly uses solar energy as energy and air and water (H_2_O) as raw materials to produce ammonia gas, avoiding the disadvantages of natural gas as a feedstock for hydrogen, where hydrogen molecules can be obtained from water molecules. In addition, there is no carbon dioxide emissions in the photocatalytic nitrogen fixation, which is an ideal environmental protection nitrogen fixation technology [77,78,79]. 

**Figure 4:**
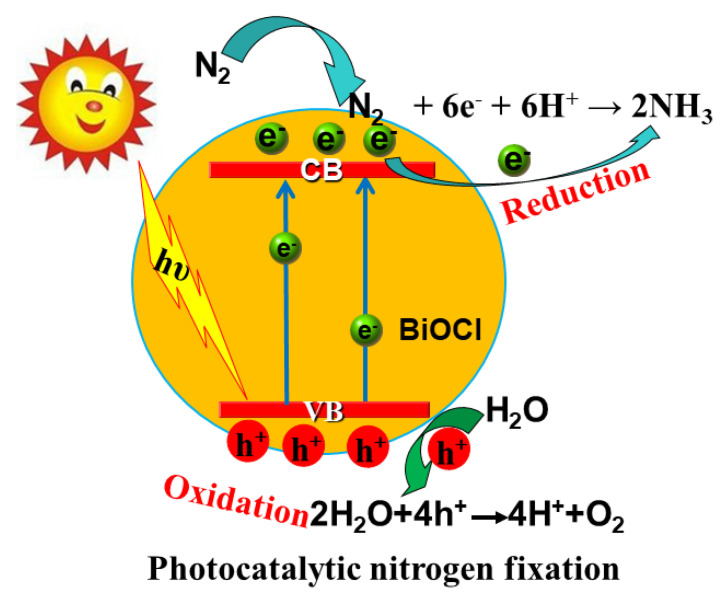
Schematic diagram of mechanism of photocatalytic nitrogen fixation over BiOCl photocatalytic.

In 1997, Schrauzer and Guth conducted the first photocatalytic nitrogen fixation study by using titanium dioxide photocatalysts under UV light irradiation [80]. Since then, especially in the 21st century, a lot of research work has been carried out to explore various applicable catalysts and improve the performance of photocatalysts for nitrogen reduction [81]. In the exploration process, researchers found that BiOX is a potential and promising photocatalytic nitrogen fixation catalysts. The reaction of photocatalytic reduction of nitrogen over BiOX photocatalysts can be divided into the following steps (Figure 4) [82]: (1) nitrogen adsorption, the surface-active site of BiOX to fix nitrogen; (2) BiOX uses the captured light energy to produce photogenerated electrons. The photogenerated electrons migrate to the conduction band, leaving holes in the valence band; (3) Some electrons combine with holes, and some electrons and holes migrate to the surface of the BiOX to participate in the REDOX reaction; (4) H_2_O can be oxidized to produce oxygen gas via the holes, while nitrogen is reduced to ammonia after a series of multi-step injection of photogenerated electrons and water-derived protons.

In fact, pure BiOX exhibited low performance of photocatalytic nitrogen fixation. Therefore, many researchers have proposed various modification strategies to improve its catalytic activity. For example, Li et al. prepared the surface oxygen vacancies on BiOBr nanosheets, which could effectively increase the N_2_ adsorption and activate the inert nitrogen molecules, while facilitating the efficient separation of photoelectrons and holes [83]. Therefore, the photocatalytic activity of nitrogen fixation was greatly improved. Xue et al. reported that the photocatalytic nitrogen fixation performance of BiOBr was improved about 10 times by the synergistic effect of oxygen vacancy and ultra-thin layer structure [84]. Gao et al. loaded the flower-like BiOBr onto the inner and outer sides of the C_3_N_4_ nanotubes simultaneously, effectively realizing the separation of photogenerated electrons and hole pairs, and thus increasing the photocatalytic nitrogen fixation activity of BiOBr by 13.9 times [85]. Now, photocatalytic nitrogen fixation capacities of BiOX are still unlikely to replace the Haber–Bosch process, but their potentials are huge and need further exploration.

### 3.4. Degrading of Inorganics (Hexavalent Chromium Ions)

Hexavalent chromium ion (Cr(VI)) is highly toxic, easy to cause cancer, and even causes gene mutations. It cannot be biodegraded into harmless substances, resulting in enrichment in the H_2_O, ultimately endangering human health [86,87]. However, trivalent chromium ions (Cr(III)) do not easily enter cells, so they are generally considered almost non-toxic. Therefore, reducing hexavalent chromium is particularly important, even imminent [88,89]. 

**Figure 5:**
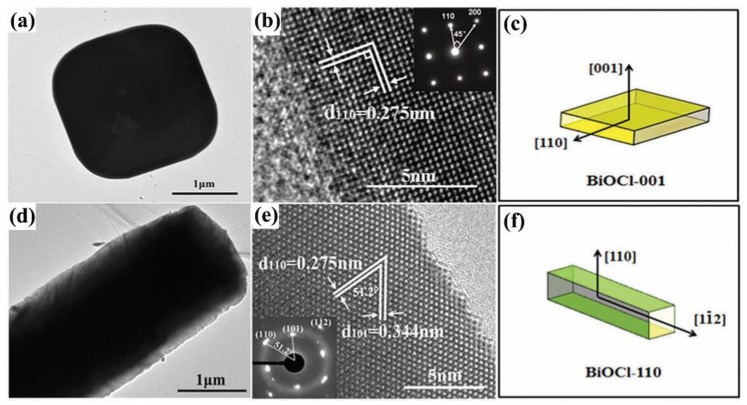
TEM, HRTEM images, and crystal facet models of BiOCl-001 (**a**–**c**) and BiOCl-110 (**d**–**f**). Reprinted with permission from Ref. [93]. Copyright 2018, The Royal Society of Chemistry.

Recently, BiOX is useful for the photocatalytic reduction of hexavalent chromium ions [90,91]. In BiOX photocatalytic system, Cr(VI) has a strong oxidizing ability and could be considered an electron trapping agent, thereby being reduced by the photogenerated electrons. For example, Fan et al. prepared the BiOBr nanoflowers of high exposure (110) facets, which showed an excellent photocatalytic removal capacity for Cr(VI). The result exhibited that the whole reaction for Cr(VI) reduction was only 50 min [92]. To investigate the effect of different crystal facets of BiOCl on the photocatalytic reduction for Cr(VI) in detail, Peng et al. synthesized BiOCl with exposed (110) and BiOCl with exposed (001) facets samples (Figure 5), respectively. They found that BiOCl-110 has more excellent photoreduction activity compared to that of BiOCl-001, and 40 mL of Cr(VI) (30 mg/L) could be completely reduced within 10 min under neutral conditions [93]. To overcome the problems of insufficient light absorption capacity and low separation efficiency of photogenerated carriers, Hussain et al. prepared BiOCl_0.8_Br_0.2_ solid solution, which exhibited visible light absorption ability, a significant increase in light absorption relative to pure BiOCl [94]. At the same time, the solid solution structure is very favorable for the efficient separation of photogenic carriers. Therefore, BiOCl_0.8_Br_0.2_ exhibited a better photocatalytic reduction activity of Cr(VI) than pure BiOCl.

In addition, to further improve the activity of BiOX photocatalytic reduction of Cr(VI), researchers have conducted a lot of exploration and research, mainly focusing on the construction of heterojunction, the construction of oxygen vacancy, doping design, etc. [95,96,97]. Through the review and analysis of recent studies, the BiOX series of catalysts can be considered a class of photocatalysts with good application prospects for hexavalent chromium ion treatment.

### 3.5. Reducing Carbon Dioxide to Organic Carbon Resources

Human activities have caused a large amount of carbon dioxide emissions, global warming is a serious problem, is also one of the most pressing challenges facing the world [98]. Over the past few decades, people worldwide have been increasingly exposed to extreme weather hazards caused by global warming, such as storms, floods, and droughts. Therefore, reducing carbon dioxide emissions, using CO_2_ reuse, and achieving sustainable development have become a consensus. Harnessing solar energy to convert CO_2_ into valuable chemicals and fuels is considered one of the effective solutions to global warming and energy needs [99,100]. 

One of the key factors affecting the activity and selectivity of photocatalysts is the separation efficiency of its photogenerated carriers in the photocatalytic CO_2_ reduction process. However, conventional photocatalysts face the problem of easy recombination of photogenerated carriers, resulting in low electron transfer efficiency, so the performance of CO_2_ photoreduction is not ideal. Recently, it has been shown that two-dimensional nanomaterials show good photocatalytic carrier separation efficiency when used for the photoreduction of CO_2_. BiOX series photocatalysts have unique two-dimensional layered structures, which are considered promising materials for the photocatalytic reduction of CO_2_. Their unique layered structures could improve the separation of photogenerated carriers and facilitates electron migration to the surface-active site, thereby accelerating the photocatalytic CO_2_ reduction reaction. Therefore, carbon dioxide reduction of BiOX series photocatalysts has become a hot topic [101,102,103].

**Figure 6:**
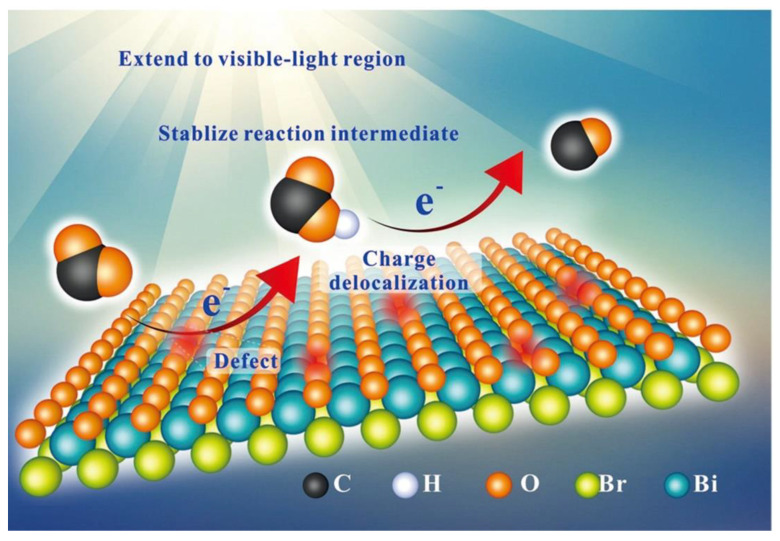
Schematic diagram of mechanism of CO_2_ photoreduction over the BiOBr atomic layers. Reprinted with permission from Ref. [102]. Copyright 2018, Wiley.

For example, Wu et al. prepared BiOBr atomic layers with many oxygen vacancies, which exhibited a CO generating rate of 87.4 umol g^−1^ h^−1^ in the process of visible-light-driven CO_2_ reduction [104]. Via in situ FTIR and DFT calculations, they proposed a possible reaction path (Figure 6), which is as follows: (1) firstly, CO_2_ and H_2_O are adsorbed on the BiOBr surfaces. Subsequently, H_2_O adsorbed on the BiOBr surface will be dissociated into hydrogen and hydroxy ions. CO_2_ will be transferred into CO_2_* active species. (2) Additionally, then, the CO_2_* adsorbed BiOBr surfaces react with the surface protons, leading to the formation of a COOH* intermediate. (3) A COOH* intermediate protonation process is carried out to produce the CO* molecules. (4) The CO* active species will desorb from the BiOBr surface to form the final CO molecule. To further restrain the photogenerated electron-hole pair recombination rate and improve the product yield of BiOX photocatalysts, Sun et al. proposed to construct an effective heterojunction [105]. Therefore, they prepared an In_2_O_3_/BiOI composite, which showed 5.3 times higher yields of CO than those of pure BiOI. This can be attributed to forming a type II heterojunction, which promotes efficient charge separation and transfer at the heterojunction interface.

In the past few decades, researchers have made much important progress in the CO_2_ reduction of BiOX photocatalytic nanomaterials. The unique layered structure of BiOX provides a good possibility for displaying excellent photocatalytic CO_2_ reduction activity, but fewer active centers and low photogenerated electron transport efficiency are still not ideal. In recent years, to further improve the transport efficiency of photogenerated electrons and holes, many researchers have made BiOX into ultra-thin nanosheets and nanotubes, doped elements, and constructed surface Lewis’s acid-base pairs on the surface of BiOX, and so on [106,107,108]. These strategies have greatly improved the photocatalytic CO_2_ reduction performance of BiOX. However, there is still a huge gap between the efficiency of photocatalytic CO_2_ reduction of BiOX and the actual production level, which requires further exploration and research.

### 3.6. Killing Bacteria

In our living environment, bacteria are everywhere, among which, the breeding of harmful bacteria will induce human diseases and harm human health. Therefore, how to solve harmful bacteria more effectively is a hot topic in international research. Currently, photocatalysis technology is considered a low-cost and environmentally friendly way to efficiently inactivate various bacteria [109,110,111,112,113]. Additionally, BiOX is a series of new types of photocatalytic materials, which is considered one of the most promising antibacterial materials due to its unique layered crystal structure, suitable band gap, high chemical stability, long-lasting antibacterial effect, low price, safety, and other advantages [114,115,116].

For example, Attri et al. prepared Ni-doped BiOCl nanosheets, which was observed Ni-BiOCl exhibited excellent photocatalytic antibacterial activity against *S. aureus* bacteria under visible light [117]. In the case of light conditions, the killing rate of *S. aureus* was 99.5%. They found that various active species (such as H^+^, 🞄O_2_, and OH˙ generated by the Ni-BiOCl photocatalyst) encounter the surface of bacteria, and oxidize the cell wall, disturbing the cell permeability. The above-mentioned process results in the loss of intracellular components, and biomolecules, and is responsible for cell death. Our group has prepared ultrathin nanosheets of BiOI (h-BiOI) with a thickness of about 2 nm [118]. Due to the quantum size effect, the valence band of h-BiOI is a positive shift relative to the block BiOI structure, and the oxidation capacity is significantly improved. Therefore, the bactericidal capacity of h-BiOI on Escherichia coli is significantly improved compared to block BiOIs.

The bactericidal principle of BiOX series photocatalysts is as follows: a series of active species (such as h^+^, 🞄O_2_^−^, H_2_O_2_, 🞄OH) are generated after performing REDOX reaction between BiOX and oxygen or water after light exposure. These active species can interact with biological macromolecules (for example, lipid, protein, enzymes, and nucleic acid macromolecules), directly or through a series of oxidative chain reactions to biological cells to cause damage, to achieve the purpose of sterilization. 

## 4. Future Perspectives and Summary

With the rapid consumption of underground fossil energy, the energy crisis and environmental pollution are becoming more serious. Semiconductor photocatalysis is considered a very good and promising solution to solve the environmental challenge. The key core of photocatalysis is a suitable photocatalyst. Among the many photocatalysts, the BiOX series of catalysts are considered a range of potential material due to their various advantages and was widely studied. After decades of scientific research, much research has been done to further extend the photocatalytic application field of BiOX and improve the photocatalytic efficiency of BiOX for meeting future practical processes. In this review, we summarized a relatively comprehensive six recent applications of BiOX in photocatalytic reactions. Additionally, these application mechanisms and the progress of these applications have been described briefly. Although there are decades of exploration of BiOX’s various applications and these signs of progress are good, some problems or challenges need further study or optimization, thereby promoting their future practical application. 

(1) In the field of photocatalytic splitting of H_2_O, the conduction positions of the BiOX series of catalysts are not dominant, and it is necessary to further explore some effective ways to improve their conduction position or reduce the reduction potential through activating H_2_O molecules using some modification to BiOX. 

(2) Through the continuous exploration of scientific researchers, BiOX should have relatively good activities at degrading organics and hexavalent chromium ions. However, the corresponding industrial application technology or recycling method still needs further exploration. In particular, we should try to cooperate with some relatively mature methods in the industry, such as chemical treatment of pollutants, biodegradation of pollutants, etc.

(3) Now, the capacities of photocatalytic nitrogen fixation and CO_2_ reduction of BiOX are still low and do not meet the actual industrial production. Therefore, further in-depth exploration and research are very necessary. Additionally, a reasonable modification strategy is very key. Reasonable modification strategies need to be designed based on reliable reaction mechanisms. Therefore, in the future, we first need to conduct a detailed exploration of the complex mechanism of photocatalytic nitrogen fixation and carbon dioxide reduction. In addition, I think it is also important to design some synergistic strategies in the process of photocatalytic CO_2_ reduction, such as photothermal synergistic catalysis and the synergy of emerging surface frustrated Lewis’s acid-base theory with existing modification strategies.

(4) The photocatalytic antibacterial research of BiOX series catalysts is of great significance. Photocatalytic technology can effectively avoid antibiotic resistance caused by using antibiotics. The BiOX series catalysts’ recovery and lack of diffusion capacities during antibacterial use are crucial limiting factors. In future studies, we need to combine BiOX series catalysts with some suitable carriers to overcome the above problems.

## Figures and Tables

**Table 1:** These photocatalytic applications of BiOX.

BiOX Six Applications in the Photocatalytic Field
Photocatalytic CO_2_ reduction
Photocatalytic degrading inorganics
Photocatalytic killing bacteria
Photocatalytic N_2_ fixation
Photocatalytic degrading organics
Photocatalytic splitting H_2_O

## Data Availability

Data will be made available on request.
